# Analysis of Oral Health Status and Dental Caries-Related Factors in Children of Zhoushan

**DOI:** 10.3290/j.ohpd.c_2029

**Published:** 2025-06-03

**Authors:** Songtao Pan, Di Miao, Yingna Xu, Chenting Xin

**Affiliations:** a Songtao Pan Dentist, Department of Stomatology, Zhoushan Stomatological Hospital, Zhoushan 316000, Zhejiang, China. Designed and conducted the research study; wrote the manuscript.; b Di Miao Dentist, Department of Stomatology, Zhoushan Stomatological Hospital, Zhoushan 316000, Zhejiang, China. Designed and conducted the research study; wrote the manuscript.; c Yingna Xu Dentist, Department of Stomatology, Zhoushan Stomatological Hospital, Zhoushan 316000, Zhejiang, China. Designed and conducted the research study; wrote the manuscript.; d Chenting Xin Dentist, Department of Stomatology, Zhoushan Stomatological Hospital, Zhoushan 316000, Zhejiang, China. Designed and conducted the research study; wrote the manuscript.

**Keywords:** children, related factors, dental caries, oral health, diet habits, oral hygiene practices, intake of desserts or sweetened beverages

## Abstract

**Purpose:**

This study was initiated to assess the oral health status and identify factors associated with dental caries in children from Zhoushan.

**Methods and Materials:**

A total of 125 children aged 5–12 years old were selected for oral examinations at the paediatric dentistry department of our hospital. The dental caries status of the children was examined and recorded, and a questionnaire was administered to their caregivers. The questionnaire collected data on patients’ awareness of oral health, supervision of children’s toothbrushing, assessment of brushing effectiveness, as well as dietary habits, oral behaviours, and oral healthcare practices. Univariate and multivariate logistic regression analyses were employed to analyse the relationships between the prevalence of dental caries and the selected variables.

Results: The caries rate among the 125 children was 62.40%. Logistic regression analysis showed that the use of fluoride toothpaste, brushing ≥ 2 times per day, and brushing after eating were protective factors against dental caries in children (OR < 1, P < 0.05). In contrast, age at initiation of brushing > 3 years, consumption of sweets and beverages ≥ 2 times per week, and frequent bedtime eating were likely to increase dental caries risk in children (OR > 1, P < 0.05).

**Conclusion:**

In view of the identified factors contributing to dental caries in children, it is important to strengthen oral hygiene education for both parents and children. Promoting standardised dental caries prevention and treatment practices and cultivating good oral hygiene habits among children are essential for improving their oral health.

Dental caries is a biofilm induced disease disease that can spread and cause prolonged low pH levels in the oral cavity, leading to a net loss of minerals from teeth. ^10^ It remains a global healthcare challenge with significant economic impacts and uneven distribution across countries. ^2^ According to the World Health Organization (WHO), 60–90% of children are influenced by dental caries, which impacts individuals of all age groups, although children are more susceptible than adults. ^9^ If left untreated, dental caries in children can lead to toothaches and difficulties in eating or sleeping, thereby hurting their daily activities. Meanwhile, untreated dental caries in children also negatively impact their families. ^1^ Dental caries results from the complicated interactions between fermentable carbohydrates, acid-producing bacteria, and various internal and external host factors over time. The risk factors of dental caries includes biological, physical, social, environmental, and behavioural characteristics, as well as factors associated with living conditions and lifestyle. ^4^ Various organic (proteins and peptides) and inorganic (water and electrolytes) components help protect teeth from dental caries. These protective mechanisms function through processes such as clearing food residues and sugars, aggregating and eliminating microorganisms, buffering acid, maintaining the supersaturation of tooth minerals, forming acquired films, as well as providing antibacterial defence. ^6^ In epidemiology and clinical practice, identifying risk factors for caries is crucial for developing effective prevention strategies at both individual and collective levels. ^12^ Children aged 6–12 years are in the mixed dentition stage, while those aged 12–15 years are in the young permanent dentition stage, where deciduous teeth are replaced with permanent ones. This transitional period presents an opportunity for the replacement of milk caries, making effective intervention crucial for their oral health and overall development in the future. Herein, this study was launched to analyse the oral health status and dental caries-related factors in children of Zhoushan, providing a reference for the targeted implementation of children’s oral healthcare initiatives.

## MATERIALS AND METHODS

### Ethical Approval

This study was ratified by the ethics committee of the Zhoushan Stomatological Hospital WPS_1665901006. The informed consent form was signed by the parents or guardians of all enrolled children.

### General Information

This was a cross-sectional study. A total of 125 cases of children who underwent oral examinations in the paediatric dentistry department of our hospital from January to December 2022 were included. The inclusion criteria for patients were: children aged 5 to 12 years old.; those without serious heart, brain, and kidney diseases; residents of the area; and those without congenital malformations of the oral and maxillofacial region, whose guardians provided informed consent. Exclusion criteria included poor compliance and inability to communicate; impairments in vision, hearing, or writing that hindered communication, situations where accompanying personnel were not immediate family members, and cases where the questionnaire could not be completed for various reasons.

### Methods

Two paediatric dentists, each with more than 5 years of clinical experience, conducted the oral examinations. Both dentists passed a standard consistency test with a Kappa value greater than 0.8. The examinations were conducted according to the World Health Organization (WHO)’s *Basic Methods for Oral Health Surveys* (4th edition). The specific indicators assessed included caries in deciduous teeth, caries in permanent teeth, pit and fissure sealing, and dental fillings. Caries were diagnosed based on WHO diagnostic criteria, including the presence of visible cavities, visible under-enamel destruction, or detectable lesions in the grooves or smooth surfaces of teeth, excluding physiological loss of deciduous teeth or damage from physical actions such as chipping or missing teeth. This diagnosis also included filled or missing caries.

Questionnaire survey: A questionnaire was designed based on the ‘Fourth National Oral Health Epidemiological Survey Scheme’, combined with expert opinions and preliminary research findings, to investigate children’s knowledge and behaviours related to oral health. Trained surveyors distributed the questionnaires, provided detailed instructions to parents on how to complete them, and clarified any questions regarding the content. The questionnaire included sections on the child’s name, age, gender, parents’ literacy, awareness of oral health knowledge, supervision of toothbrushing, effectiveness of brushing, and dietary habits, oral behaviours, oral health attitudes, and oral healthcare practices. A total of 125 questionnaires were distributed and collected, with no invalid responses, yielding a 100% effective response rate. To assess consistency, 20 parents were randomly selected for follow-up by telephone, resulting in a 95% concordance rate. Surveyors were trained in non-directive survey techniques, and all passed a standard consistency test with a Kappa value above 0.8. Two individuals independently entered the data both the questionnaires and oral examinations into EXCEL, followed by dual-entry verification and immediate comparison and correction to ensure data reliability.

### Statistical Analysis

Data were processed using SPSS 24 software (IBM Corp, Armonk, NY, USA). The Chi-square test was adopted for categorical data, which were expressed as a percentage (%). Multivariate logistic regression analysis was utilised to identify factors related to dental caries in children. Differences were considered statistically significant when P < 0.05.

## RESULTS

### Basic Information

Following oral examination, 78 out of 125 children were found to have dental caries, resulting in a caries rate of 62.4%. Among these, 42 boys and 36 girls were affected by caries. The caries rate was 58.97% for children at 5–8 years old and 41.03% for children at 9–12 years old. There was no notable difference between genders (P = 0.070) and ages (P = 0.274) in terms of the caries rate (Table 1).

**Table 1:** Basic information of children

	Suffering from dental caries (n = 78)	Not suffering from dental caries (n = 47)	P
Gender			0.070
Boy	42 (53.85)	33 (70.21)	
Girl	36 (46.15)	14 (29.79)	
Age (years)			0.274
5–8	46 (58.97)	23 (48.94)	
9–12	32 (41.03)	24 (51.06)	


### Relationship Between Parental Factors and Children With Dental Caries

Statistical analysis revealed no significant difference in the education level of parents (P = 0.194), whether parents received health education (P = 0.060), whether they supervised their children’s brushing daily (P = 0.171), whether they brought their children for regular dental checkups (P = 0.212), or their knowledge of oral health (P = 0.237) in relation to the presence of dental caries in children. However, there was a significant association between the use of fluoride toothpaste and the occurrence of dental caries (P < 0.001) (Table 2).

**Table 2:** The relationship between parents of children and children with dental caries

	Suffering from dental caries (n = 78)	Not suffering from dental caries (n = 47)	P
Parents’ years of education (years)			0.194
< 9	25 (32.05)	10 (21.28)	
≥ 9	53 (67.95)	37 (78.72)	
Parents receiving health education			0.060
Yes	22 (28.21)	21 (44.68)	
No	56 (71.79)	26 (55.32)	
Parental supervision of daily toothbrushing			0.171
Yes	40 (51.28)	30 (63.83)	
No	38 (48.72)	17 (36.17)	
Parents’ acquisition of oral knowledge			0.237
Yes	33 (42.31)	31 (53.19)	
No	45 (57.69)	16 (46.81)	
Parents take children for regular checkups			0.212
Yes	20 (25.64)	17 (36.17)	
No	58 (74.36)	30 (63.83)	
Types of toothpaste for children			<0.001
Fluorinated	26 (33.33)	39 (82.98)	
Non-fluorinated	52 (66.67)	8 (17.02)	


### Relationship Between Children’s Behaviour and Dental Caries

The statistical analysis revealed significant associations between the presence of dental caries and several behavioural factors, including: age at which children started brushing their teeth (P < 0.001), frequency of brushing per day (P < 0.001), frequency of consuming sweets (P < 0.001) and sugary beverages per week (P < 0.001), bedtime eating habits (P < 0.001), brushing after meals (P < 0.001) (Table 3).

**Table 3:** The relationship between children’s behaviour and dental caries

	Suffering from dental caries (n = 78)	Not suffering from dental caries (n = 47)	P
Age of initiation of toothbrushing (years)			<0.001
≤ 3 = 0	28 (35.90)	32 (68.09)	
> 3 = 1	50 (64.10)	15 (31.91)	
Number of times per day for toothbrushing			<0.001
≤ 1	60 (76.92)	15 (31.91)	
≥ 2	18 (23.08)	32 (68.09)	
Number of times per week for eating sweets			<0.001
<2	18 (23.08)	38 (80.85)	
≥ 2	60 (76.92)	9 (19.15)	
Number of times per week for drinking beverages			<0.001
<2	24 (30.77)	36 (76.60)	
≥ 2	54 (69.23)	11 (23.40)	
Frequent bedtime eating			<0.001
Yes	57 (73.08)	8 (17.02)	
No	21 (26.92)	39 (82.98)	
Brushing after eating			<0.001
Yes	22 (28.21)	40 (85.11)	
No	56 (71.79)	7 (14.89)	


### Results of Multivariate Logistic Regression Analysis

A multivariate logistic regression analysis was performed using the factors with statistical significance U (P < 0.05) from the univariate analysis in Tables 1 to 3 as independent variables, with the presence of dental caries as the dependent variable. Protective factors for caries included the use of fluoride toothpaste (OR = 0.144, P = 0.016), brushing ≥ 2 times per day (OR = 0.207, P = 0.048), and brushing after meals (OR = 0.061, P = 0.001). Risk factors for dental caries included starting toothbrushing after age 3 (OR = 5.612, P = 0.031), consumption of sweets and beverages ≥ 2 times per week (OR = 7.892, P = 0.008; OR = 5.699, P = 0.027), and frequent bedtime eating (OR = 9.381, P = 0.005) (Tables 4 and 5).

**Table 4:** Assignment table

Variable	Assignment
Use of fluoride toothpaste	No = 0, Yes = 1
Age of initiation of toothbrushing	≤ 3 = 0, > 3 = 1
Number of times per day for toothbrushing	≤ 1 = 0, ≥ 2 = 1
Number of times per week for eating sweets	< 2 = 0, ≥ 2 = 1
Number of times per week for drinking beverages	< 2 = 0, ≥ 2 = 1
Frequent bedtime eating	No = 0, Yes = 1
Brushing after eating	No = 0, Yes = 1


**Table 5:** Results of multivariate logistic regression analysis

Factor	B	S.E.	Wald	P	Exp(B)	95% C.I.for EXP(B)
Lower	Upper
Types of toothpaste for children	–1.940	0.802	5.853	0.016	0.144	0.030	0.692
Age of initiation of toothbrushing	1.725	0.799	4.666	0.031	5.612	1.173	26.843
Number of times per day for toothbrushing	–1.575	0.795	3.924	0.048	0.207	0.044	0.983
Number of times per week for eating sweets	2.066	0.774	7.128	0.008	7.892	1.732	35.958
Number of times per week for drinking beverages	1.740	0.789	4.862	0.027	5.699	1.213	26.772
Frequent bedtime eating	2.239	0.806	7.715	0.005	9.381	1.933	45.535
Brushing after eating	–2.789	0.806	11.987	0.001	0.061	0.013	0.298


## DISCUSSION

Dental caries is a common disease affecting children worldwide, with an estimated prevalence of around 50%. If left untreated, it can impair chewing function, negatively impacts a child’s smile, speech, psychosocial environment, as well as overall quality of life for both children and their families. ^13^ Dental caries is a multifactorial condition influenced by physical, biological, behavioural, environmental, as well as lifestyle factors, including insufficient saliva flow, high levels of cariogenic bacteria, poor oral hygiene, second-hand smoke exposure, improper infant feeding practices, along with socioeconomic status. The development of dental caries primarily results from the interaction of oral bacteria, host, diet, and time. ^17^

In this study, we found no statistically significant differences in caries rates between gender and age groups. This contrasts with findings from other studies, such as those by Soraya Zahmatkesh, Selamawit Bassa, and Trudy Voortman, which report higher caries rates in children aged 2-6, 6-12, and 13 years old. ^3,14,18^ The discrepancy could be due to differences in sample size and geographical characteristics. Additionally, we also uncovered that the use of fluoride toothpaste, brushing ≥ 2 times per day, and brushing after meals were protective factors for dental caries in children. Conversely, starting brushing after the age of 3, consumption of sweets and beverages ≥ 2 times per week, and frequent bedtime eating were likely to increase caries risk in children.

As societal changes evolve, the factors influencing dental caries in children have become increasingly complex. While the specific factors vary across regions, sweet and sugary beverage consumption and oral hygiene habits remain key determinants of caries. ^16,20^ The intervention targeting children’s dietary and hygiene behaviours can effectively reduce the incidence of dental caries. The frequent intake of sweets and sugary foods leads to sugar adhering to tooth surfaces, where oral bacteria decompose the sugar to produce acid, contributing to tooth corrosion. ^7^ Therefore, it is recommended that that children should limit their intake of sweets and sugary beverages to no more than three times a week. They should also prioritise foods rich in fibre or low in sugar. After eating, it’s important to rinse their mouths, brush their teeth, floss, and take other measures to prevent food residue buildup and promote oral hygiene. Fluoride plays an essential role in the prevention of dental caries by enhancing tooth mineralisation and bone density, having bactericidal effects on caries-causing bacteria, and inhibiting demineralisation while promoting remineralisation of enamel when present in plaque and saliva. ^8^ A meta-analysis has confirmed that the use of standard fluoride toothpaste is effective in preventing both milk caries and permanent caries. ^5^ Fluoride toothpaste reduces enamel and dentin dissolution due to acid, inhibits demineralisation, and strengthens remineralisation. Encouraging the use of fluoride toothpaste is a self-care strategy to prevent caries. In the future, Zhoushan City should increase fluoride toothpaste promotion, emphasising its importance in community schools and, if necessary, offering free distribution.

This study identified multiple factors influencing dental caries in children, which align with findings from other studies that report regional and cultural variations. For instance, in low- and middle-income countries, high sugar intake, low maternal education, and both low and high socioeconomic status increase the risk of dental caries. Conversely, good brushing habits, higher maternal education, assistance with brushing, and middle socioeconomic status serve as protective factors. ^19^ Other research has also linked low body mass index in children aged 3 to 6 years with a higher risk of caries. ^11^ Shahram Mosharrafian et al. also supported that children exposed to passive smoking were found to have a higher incidence of dental caries, likely due to increased plaque accumulation and the adverse effects on oral health from passive smoking. ^15^ These findings underscore the need for further research to comprehensively assess the factors influencing children’s oral health and caries risk.

In summary, the oral health status of children in Zhoushan City is concerning, with numerous risk factors contributing to the high prevalence of dental caries. Targeted preventive measures should be implemented in clinics to improve the oral health of children in the region. Health education interventions should focus on improving children’s understanding of oral hygiene, instructing them on correct brushing techniques, and promoting healthy dietary habits to reduce caries incidence. Parents should be encouraged to limit their children’s intake of sweets and carbonated beverages, avoid bedtime eating, encourage the use of fluoride toothpaste, ensure regular dental checkups, and strengthen health education. However, this study still has some limitations. For instance, no sample size calculation was conducted, and the sample size was relatively small. Furthermore, since all participants were sourced from the paediatric dentistry department of a single hospital, this may introduce bias in the study results due to the sample’s lack of representativeness. Given that Zhoushan may have unique factors influencing children’s oral health, the generalizability of these results to other regions may be limited. Therefore, future studies should include a larger, more diverse sample to improve the external validity of the findings.
